# Toxocarosis in children: poor hygiene habits and contact with dogs is related to longer treatment

**DOI:** 10.1007/s00436-018-5833-7

**Published:** 2018-03-20

**Authors:** Anna Kroten, Kacper Toczylowski, Elzbieta Oldak, Artur Sulik

**Affiliations:** Department of Pediatric Infectious Diseases, Medical University of Bialystok, Waszyngtona 17, 15-274 Bialystok, Poland

**Keywords:** Toxocarosis, Toxocara, Children, Dogs

## Abstract

The objective of this study was to investigate the main clinical signs and symptoms of toxocarosis in children and the treatment results. The study group consisted of 66 seropositive children aged 2 to 16 years, evaluated in an outpatient clinic in north-eastern Poland for 24 months. Male gender and living in urban areas predominated in the study population. Children presented with non-specific symptoms, of which the most common was abdominal pain or tenderness, which was reported by 39 (59%) patients. Absolute eosinophil counts were increased in 32 (48%) children. Total IgE concentrations were increased in 31 of 55 (56%) tested children. All evaluated children received albendazole as a first-line treatment. In 19 cases, additional treatment with albendazole and/or diethylcarbamazine was provided. The analysis of possible causes of prolonged treatment revealed that significant risk factors were geophagia [odds ratio (OR), 6.3; 95% confidence interval (95% CI), 1.8–21.8; *p* < 0.01] and daily contact with a dog [OR, 5.9; 95% CI, 1.3–27.3, *p* < 0.05]. We hypothesise that poor hygiene habits and daily contact with a dog pose a risk of reinfection and limits treatment efficiency. Because of non-specific signs and frequent lack of eosinophilia, physicians should maintain high levels of suspicion for toxocarosis, particularly in patients who live in regions heavily contaminated with *Toxocara* eggs.

## Introduction

Toxocarosis is a helminth zoonosis caused by ascarid larvae of the *Toxocara* genus, which are common in domestic dogs and cats. The increased number of dogs and cats in human environments and negligence of basic hygiene and prophylactics increase exposure in humans. Humans acquire the parasite by ingesting infective eggs, which are usually found in soil contaminated by dog or cat faeces. The disease may also be contracted by accidental ingestion of eggs from unwashed fruits and vegetables, by ingesting raw or undercooked meat that contains larvae, or from animal fur (Macpherson 2013; Thomas and Jeyathilakan 2014; Borecka and Kłapeć 2015). Significant risk factors for *Toxocara* transmission include owning dogs and cats, onychophagia (nails biting), geophagia, living in a contaminated environment and poor socioeconomic status (Carvalho and Rocha 2011; Lötsch et al. 2016). Locations such as playgrounds and sandboxes are often accessed by animals and present high likelihood of contamination (Kroten et al. 2016). Children spend a considerable amount of time in such places and thus are exposed to *Toxocara* infections (Mazur-Melewska et al. 2012). For these reasons, most patients are children aged 2 to 8 years with a history of onychophagia, geophagia and exposure to animals (Fragoso et al. 2011; Wiśniewska-Ligier et al. 2012; Romero Núñez et al. 2013).

Parasites do not reach full maturity in the human body. Hatched larvae can penetrate the intestine and migrate to other tissues and organs via blood and lymph. The clinical manifestations of toxocarosis depend on the number of larvae ingested, duration of infection, circulation of larvae in the body and immune response of the host. The severity of infection depends on the immune system response to the parasite (Fan et al. 2013).

The types of toxocarosis are visceral, ocular, neuronal and covert (Carvalho and Rocha 2011; Mazur-Melewska et al. 2012). Visceral larva migrans (VLM) is associated with migration of larvae to the liver, lungs and other organs of the host. The clinical signs of visceral toxocarosis include cough, fever, weakness, general malaise, rash, headache, oedema, abdominal pain and hepatomegaly (Carvalho and Rocha 2011; Mazur-Melewska et al. 2012; Macpherson 2013; Lötsch et al. 2016). Neurological toxocarosis is a specific form of visceral toxocarosis and may lead to meningitis, meningoencephalitis and transverse myelitis (Caldera et al. 2013). Epidemiological studies have proven that *Toxocara* infections provoke allergy-related syndromes, including atopic asthma and atopic dermatitis (Walsh 2011; Carvalho and Rocha 2011). Ocular larva migrans (OLM) occurs when the circulating larvae migrate to the retina causing granulomatous reactions. Ocular toxocarosis typically appears unilaterally in children and young adults (Fillaux and Magnaval 2013; Ahn et al. 2014). Patients with covert toxocarosis usually present with non-specific symptoms, that together form a discernible symptom complex including fatigue, nausea, abdominal pain, headache and sleep or behaviour disturbances (Taylor et al. 1988). The diagnosis of covert toxocarosis is achieved by serology, increased blood eosinophil counts giving a first indication (Mazur-Melewska et al. 2012; Fillaux and Magnaval 2013). High eosinophilia and increased concentration of total immunoglobulin E (IgE) are hallmarks of exposure to *Toxocara*. The diagnosis of toxocarosis is usually difficult because the clinical status of this zoonosis is non-specific. The method of choice for diagnosis is the enzyme-linked immunosorbent assay (ELISA) test, which uses the excretory-secretory antigens of infective *Toxocara canis* larvae (TES) (Fillaux and Magnaval 2013; Macpherson 2013). Western blot (WB) analysis is recommended for the accurate confirmation of toxocarosis.

The aim of this study was to analyse the main clinical signs and symptoms of toxocarosis in children and treatment results.

## Material and methods

This study included 66 seropositive children aged 2 to 16 years, evaluated in the Outpatient Clinic of Bialystok Children’s Clinical Hospital at the Medical University. The study sample included 31 (47%) girls and 35 (53%) boys. Thirty-six (55%) children lived in urban areas and 30 (45%) in the countryside. The average age of the study sample was 8.8 ± 3.6 years. The symptoms of toxocarosis in children were analysed after dividing the sample into two subgroups: preschool children (*n* = 18) and school children (*n* = 48). Considering age differences, preschool children tend to play more extensively in sandboxes and pay less attention to hand hygiene compared to school children. All patients were assessed for 24 months.

The study protocol was approved by the Research Ethics Committee of our institution, and informed consent was obtained from the children’s parents or legal guardians. The clinical diagnosis of toxocarosis was confirmed by the presence of specific IgG antibodies to TES using ELISA (NovaTec Immundiagnostica GmbH, Germany). The positive cut-off value was 11 NTU (NovaTec Units). In 10 patients with persistent toxocarosis-positive ELISA, the results were confirmed by WB analysis (LDBIO Diagnostics, France). Serology tests were conducted according to the manufacturer’s instructions. The presence of two or more low molecular weight bands in the range of 24–35 kDa was assumed to indicate a positive WB test. Ophthalmoscopies were done for all patients and were all negative. Our research did not include patients with the ocular form of the disease.

A questionnaire was developed to record symptoms and signs of toxocarosis. Interviews were made during the first visit to the Outpatients Clinic. Parents were asked about their children’s history of abdominal pain, lack of appetite, rashes, coughing, headaches, enlargement of lymph nodes, fever, hyperactivity, weakness, arthralgia, weight loss, muscle pain, nausea and vomiting, sleep disorders, bronchospasm and wheezing. In addition, the main risk factors for infection with *Toxocara* were investigated and recorded. Data collected on clinical examination and laboratory test results, including absolute eosinophil count, total IgE concentration and antibody titres specific to *Toxocara*, were also recorded. Eosinophilia was defined as an absolute eosinophil count of more than 450 cells/μL. Total IgE concentration was determined using immunofluorescence. The normal concentration was 33 IU/mL for children aged 2 to 4 years and 85 IU/mL for patients aged 5 to 16 years. Children were retested for *Toxocara* antibodies and eosinophilia 3 months after the first visit, following treatment. Treatment decision was made based on symptoms, physical examination, abnormalities in laboratory test results and positive serology. Children were treated with albendazole (ABZ) (15 mg/kg body weight in two divided doses daily, maximum 400 mg) for 10 days (two 5-day courses with a rest period of 10 days between each treatment). Children with persisting symptoms or high eosinophil counts after treatment were categorised as treatment failure and were given additional courses of ABZ and/or a 10-day course of diethylcarbamazine (DEC). Diethylcarbamazine was given at 6 mg/kg body weight in two divided doses daily for 10 days. Albendazole is widely available, safe and effective for treatment of toxocarosis (Magnaval and Glickman 2006). Diethylcarbamazine is considered even more effective than ABZ, but because of neurological side effects commonly associated with DEC, we did not use it as a first-line treatment.

The risk factors for treatment failure were also analysed. The significance of data was determined using parametric tests and chi-squared test. Odds ratios were calculated using multiple binominal logit regression model. A *p* value of less than 0.05 was considered statistically significant. Statistical analysis was conducted using StatSoft Statistica version 10.

## Results

### Clinical signs

The reasons for searching medical advice in the outpatient clinic in cases of suspected toxocarosis were primarily clinical symptoms (*n* = 48; 73%). Sixteen children (24%) were referred to the outpatient clinic because of abnormal results in tests conducted for other diseases, of whom four (6%) children had positive anti-*Toxocara* antibodies. Two (3%) patients were evaluated because of positive serological tests in siblings.

The majority of children presented covert toxocarosis (Table 1). The leading symptom was abdominal pain or tenderness (*n* = 39; 59%). Twenty-six (39%) children had a mild enlargement of cervical lymph nodes. Poor appetite was reported in 17 (26%) children. Other symptoms were rare. Twenty-four children (36%) reported symptoms related to VLM: rash (*n* = 12; 18%), coughing or wheezing (*n* = 11; 17%) and myalgia or arthralgia (*n* = 9; 14%). The analysis of the symptoms in the two age subgroups indicated significant differences in the rate of fever (Table 1). The results of physical examination were unremarkable in 30 (45%) of children. Four children (6%) without abnormalities in physical examination reported no symptoms but were evaluated for toxocarosis because of the accidental detection of eosinophilia (*n* = 3) and high IgE (*n* = 4).

**Table 1:** Signs and symptoms of toxocarosis in the two age subgroups

Clinical symptom	All patients (*n* = 66)	School children (*n* = 48)	Preschool children (*n* = 18)
Abdominal pain or tenderness	39 (59%)	31 (65%)	8 (44%)
Enlargement of lymph nodes	26 (39%)	18 (38%)	8 (44%)
Lack of appetite	17 (26%)	12 (25%)	5 (28%)
Rashes	12 (18%)	9 (19%)	3 (17%)
Coughing or wheezing	11 (17%)	9 (19%)	2 (11%)
Headaches	11 (17%)	10 (21%)	1 (6%)
Arthralgia or muscle pain	9 (14%)	8 (17%)	1 (6%)
Fever	8 (12%)	3 (6%)	5 (28%)*
Hyperactivity	8 (12%)	5 (10%)	3 (17%)
Weakness	8 (12%)	7 (15%)	1 (6%)
Sleep disorders	5 (7%)	4 (8%)	1 (6%)
Hepatomegaly	5 (7%)	4 (8%)	1 (6%)
Nausea or vomiting	4 (6%)	4 (8%)	0

Data presented as number of children and percentage of the group

*p < 0.05 for comparing school and preschool children

### Laboratory findings

The mean anti-*Toxocara* IgG antibody titres were 23.1 ± 10.7 NTU in the total sample and were lower in school children than in preschoolers (21.3 ± 9.9 vs. 27.8 ± 11.4, *p* < 0.05). Absolute eosinophil count was increased in 32 (48%) children. However, no significant differences were detected between the two age groups. Total IgE levels were increased in 31 of 55 tested children (56%). IgE concentrations were higher in school children (200 ± 214 IU/mL) compared with preschool children (138 ± 182 IU/mL); however, it was not possible to assess statistical differences because normal values were age dependent. In the total sample, IgE levels or eosinophilia was increased in 45 of 59 tested children (68% of the total group; data were lacking in 7 children). In the remaining 21 (32%) children, diagnosis was based on clinical diagnosis and positive serology tests.

### Treatment outcomes

**Fig. 1:**
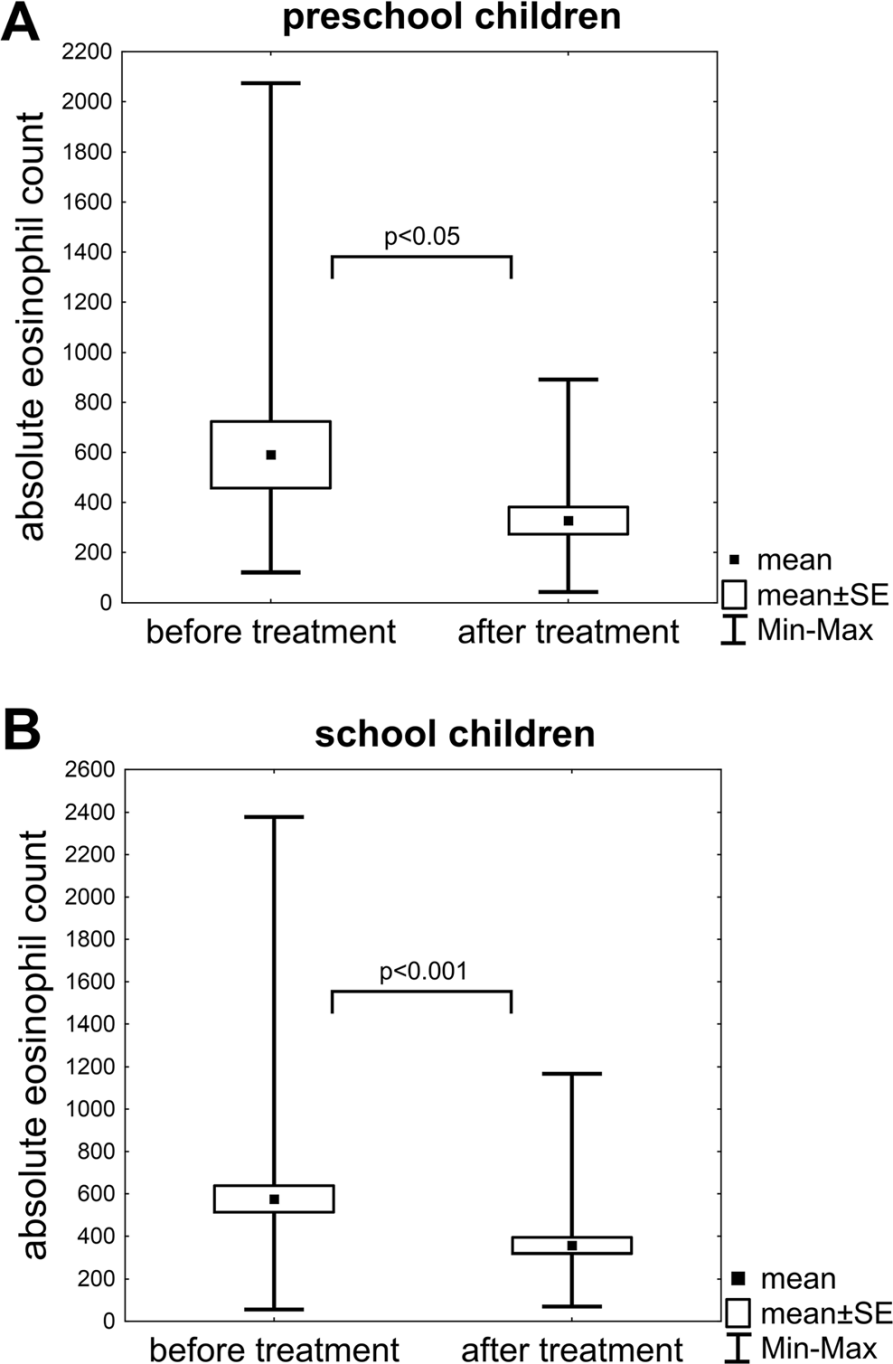
Comparison of absolute eosinophil count before and after treatment in the subgroup of preschool (**a**) and school (**b**) children

A total of 47 (71%) children were treated with a single cycle of ABZ, and 19 (29%) patients required additional treatment. Sixteen children (24%) received a second course of ABZ, and two (3%) children required more than two courses. One child received DEC as a second treatment. Two courses of ABZ and one course of DEC were necessary in one case. After treatment, specific *Toxocara* IgG antibody titres were decreased significantly in school children but not in preschoolers (Table 2). However, in the latter group, *p* value was close to the threshold (*p =* 0.054). Treatment resulted in a significant decrease of eosinophilia in both age groups (Fig. 1). Seroreversion after the first course of treatment was observed in 20 (30%) children. Four (6%) children still had positive serology 12 months after treatment, and this group had daily contact with dogs.

**Table 2:** Specific *Toxocara* IgG antibody titres

Age group	Before treatment (NTU)	After treatment (NTU)
All patients (*n* = 66)	23.1 ± 10.7	18.9 ± 15.2**
Preschool children (*n* = 18)	27.9 ± 11.4	22.3 ± 15.8
School children (*n* = 48)	21.3 ± 9.9*	17.6 ± 14.9***

Data presented as mean ± SD

*p < 0.05 for comparing titres between preschool and school children; **p < 0.01 for comparing titres before and after treatment; ***p < 0.05 for comparing titres before and after treatment

### Treatment failure

There was no difference in age, gender or place of residence among children who required additional courses of chemotherapy (Table 3). However, eosinophilia and specific antibody titres before treatment were significantly higher in this group. The analysis of risk factors revealed that daily contact with dogs and geophagia were the two most significant and independent contributors to treatment failure (Table 4). Nonetheless, it is of note that only six children were reported to eat dirt, of which two children required an additional treatment, whereas 38 children had daily contact with dogs, of which 16 received a second treatment.

**Table 3:** Comparison of children who were successfully treated with a single cycle of albendazole with children who required additional courses of chemotherapy

	Successful treatment (*n* = 47)	Treatment failure (*n* = 19)
Gender (female/male)	23 (49%)/24 (51%)	8 (42%)/11 (58%)
Place of residence (town/countryside)	28 (60%)/19 (40%)	8 (42%)/11 (58%)
Total eosinophil count before treatment (cells/μL)	498 ± 426	780 ± 522*
Total IgE levels before treatment (IU/mL)	160 ± 201	229 ± 215
Specific *Toxocara* IgG antibody titre before treatment (NTU)	20.8 ± 9.0	28.8 ± 12.6**

Laboratory findings presented as mean ± SD

*p < 0.05; **p < 0.01

**Table 4:** Analysis of risk factors associated with a need for additional treatment for toxocarosis as calculated in multiple binominal logit regression model (*n* = 19)

Risk factor	OR (95% CI)	*p*
Male gender	1.0 (0.3–3.2)	0.99
Living in a rural environments	1.2 (0.3–4.4)	0.82
Geophagia	6.3 (1.8–21.8)	< 0.01
Onychophagia or putting fingers in the mouth	0.4 (0.1–1.4)	0.13
Playing in sandboxes	1.4 (0.4–5.2)	0.58
Daily contact with dogs	5.9 (1.3–27.3)	< 0.05
Daily contact with cats	0.5 (0.1–1.8)	0.27

*OR* odds ratio, *CI* confidence interval

## Discussion

Toxocarosis is a neglected paediatric disease. Over recent years, this disease has drawn much attention because of its surprisingly high prevalence in the paediatric population. Parasite eggs are commonly found in soil and sandboxes. Poor hand hygiene habits and playing outside are the two major risk factors for contracting toxocarosis in children. As previously reported, 18 to 46% of soil and sand samples collected in our region were contaminated; however, seroprevalence was relatively low in a local paediatric population (4.2%) (Kroten et al. 2016). Previous studies reported that seroprevalence in children may vary from 1% in Spain (Guerra et al. 1995) to over 50% in Brasil (Fragoso et al. 2011) and that older age was associated with higher prevalence of antibodies against *Toxocara canis* (Mazur-Melewska et al. 2012; Romero Núñez et al. 2013, Wiśniewska-Ligier et al. 2012). Our results agree with this finding because older children accounted for 73% of the study sample. However, 18 children were younger than 7 years, indicating that children were being exposed to *Toxocara* infection early in life.

The clinical manifestations of toxocarosis are non-specific (Carvalho and Rocha 2011; Mazur-Melewska et al. 2012). Our results indicated that most patients presented with clinical signs of covert toxocarosis, including abdominal pain, cervical lymphadenopathy or loss of appetite. Of note is that abdominal pain was the most commonly reported symptom in both age groups. It is important to exclude toxocarosis in children with recurrent abdominal pain before labelling it as idiopathic (Taylor et al. 1988). Signs typical of VLM such as coughing, wheezing, myalgia or skin manifestations in this study were rare, and this result agrees with those of other studies (Ma et al. 2018). These clinical signs were not related to age, and a similar result was found by Mazur-Malewska et al. (2012). The only sign more frequently observed in younger children was fever. Fever is a non-typical sign of toxocarosis, and the difference in the rate of fever may be a coincidence because children younger than 6 years have several viral infections every year. It is more likely that fever reported by parents was associated with recent respiratory tract infection rather than toxocarosis. Of note, additional laboratory test results revealed eosinophilia or high total IgE levels in 68% of children only. The absence of eosinophilia however does not exclude toxocarosis. In a study by Taylor et al., 27% of seropositive patients had normal eosinophil counts (Taylor et al. 1988). Moreover, the results of physical examination on the day of consultation were unremarkable in 45% of children. This result demonstrates that the diagnosis of toxocarosis is challenging and requires high levels of suspicion. Therefore, physical examination and abnormal laboratory test results alone may not be good predictors of covert toxocarosis, and the analysis of patient history and epidemiological data is crucial in diagnosis.

The assessment of treatment response to *Toxocara* infection is difficult and cannot be based on clinical signs alone because these signs are non-specific. Moreover, clinicians should not rely solely on serology because antibody titres may remain high for several months despite treatment (Turrientes et al. 2011; Wiśniewska-Ligier et al. 2012). Therefore, decreased total IgE level or eosinophil count together with resolution of symptoms is used as a marker of treatment response (Elefant et al. 2006). In our study, treatment reduced total IgE levels and eosinophil counts in both age groups. Antibody levels were also decreased; however, in four children, antibody titres remained high for more than 12 months.

Albendazole and DEC are considered effective for the treatment of toxocarosis; however, in some patients, the efficacy is limited. There is a need for clinical trials evaluating novel treatment options for toxocarosis, because now we base treatment decisions on clinical studies performed in 1980s (Magnaval and Glickman 2006). In this study, we analysed why some children required additional treatment. We hypothesised that risk factors for treatment failure and infection were the same. Previous studies show that toxocarosis is more prevalent among boys (Santarém et al. 2011; Fragoso et al. 2011; Wiśniewska-Ligier et al. 2012; Romero Núñez et al. 2013). This result may be because boys tend to spend more time outdoors playing in backyards and sandboxes and thus are exposed to *Toxocara* eggs more frequently than girls. Furthermore, male gender was slightly higher in our study sample but did not significantly affect treatment outcomes.

The number of *Toxocara* spp. infections was reported to be higher in rural areas (Wiśniewska-Ligier et al. 2012). However, in the present study, living in rural environments was not associated with treatment outcomes. The rate of soil contamination with *Toxocara* eggs in Bialystok was similar to that in rural areas (Kroten et al. 2016), and this may explain the observed lack of correlation with the place of residence.

As expected, we found a positive association with geophagia, but these results should be interpreted with caution because of the small number of children with this habit. Other risk factors, including onychophagia, putting fingers in the mouth, playing in sandboxes and daily contact with cats were not related to treatment failure.

Importantly, we found that children who had daily contact with dogs were more likely to receive prolonged treatment. Contact with dogs is a relevant risk factor for contracting toxocarosis (Santarém et al. 2011; Fragoso et al. 2011; Sowemimo et al. 2017; Silva et al. 2017). It is possible but unlikely that animal fur is a direct source of infection (Keegan and Holland 2010). Dogs disseminate unembryonated eggs, which are not infectious. Therefore, pet owners are not exposed to the disease more frequently than other groups, especially if they take good care of their pets (Traversa et al. 2014; Gabrielli et al. 2017). More significant risk factors are onychophagy, geophagy and spending time outside in spaces frequented by dogs. *Toxocara* eggs disseminated in public areas gradually become infective, making these spaces suitable for toxocarosis. Therefore, it is possible that contact with dogs and spending more time in areas contaminated with *Toxocara* eggs increased the risk of reinfection in the evaluated children. Accordingly, the results of our previous study indicated that all the soil samples collected from regions where children with persisting toxocarosis lived where highly contaminated with *Toxocara* eggs (Kroten et al. 2016).

## Conclusions

Toxocarosis should be investigated in at-risk children who live in environments contaminated with *Toxocara* eggs. The diagnosis is difficult because non-typical signs are common and laboratory test results may be normal. Treatment with ABZ is usually very effective; however, the daily contact with dogs and poor hygiene habits may limit treatment efficiency.
